# Expression of circulating cell-free nucleic acids around miniscrew implant during orthodontic tooth movement—a prospective study

**DOI:** 10.1186/s40510-021-00378-0

**Published:** 2021-10-18

**Authors:** Tabassum Qureshi, Ritu Duggal, Om Prakash Kharbanda, Moganty R. Rajeswari

**Affiliations:** 1 Division of Orthodontics and Dentofacial Deformities, Centre for Dental Education and Research, All India Institute of Medical Sciences, New Delhi, 110029 India; 2 Dr CG Pandit National Chair of Indian Council of Medical Research, New Delhi, India; 3 Department of Biochemistry, All India Institute of Medical Sciences, New Delhi, 110029 India

**Keywords:** MSI, Biomarker, cfNA, Peri-implant crevicular fluid

## Abstract

**Objectives:**

Literature shows that the expression of various biomarkers in peri-miniscrew crevicular fluid (PMICF) is related to the stability of miniscrew implants (MSIs). The present study investigated the role and alterations in levels of circulating cell-free nucleic acids (cfNAs) in PMICF before and after orthodontic loading.

**Material and methods:**

This prospective study consisted of forty-six MSIs placed between the second premolar and first molar in the maxillary and mandibular arches. Direct loading was done after 3 weeks of MSI insertion with nickel-titanium closed coil spring exerting a force of 200 g. The PMICF sample was collected at various time intervals, and the level of cfNA was determined. Clinical parameters, including implant mobility and gingival health, were also assessed. Pre-loading and post-loading parameters were assessed using Wilcoxon’s rank-sum test.

**Results:**

Among 46 MSIs, 36 were stable during the study and 10 MSIs showed peri-implant inflammation and increased mobility. There was a significant rise in the cfNA concentration 24 h after implant insertion (0.4 ± 0.86 ng/μl). The level of cfNAs significantly decreased over 3 weeks and reached the baseline level (0.2 ± 0.31 ng/μl). There was also a significant rise in the levels of cfNA (0.8 ± 0.70 ng/μl) at 24 h after loading MSIs, which gradually decreased to 0.2 ± 0.24 ng/μl after 63 days. The expression of cfNAs was on the average 0.32 units more in the cases with failed implants (*P* = 0.05).

**Conclusions:**

cfNA levels in PMICF showed an upward trend 24 h after MSI insertion and 24 h after orthodontic loading. The expression of cfNA was more in cases with failed MSIs. Hence, the cfNAs can be considered as a prognostic biomarker of MSI stability.

## Introduction

The introduction of the miniscrew implant (MSI) has revolutionized the field of orthodontics [1, 2]. An MSI is a temporary anchorage device (TAD) that provides better anchorage than a conventional anchorage system [3]. An MSI is used for shorter time periods; therefore, the primary stability relies mainly on mechanical holding, whilst the secondary stability relates to the biological seal around the MSI [4, 5]. The success rate of MSIs is reported to be in the range of 57–95.3%, with an average success rate of 84% [6, 7].

The effectiveness and the clinical application of the MSI have been reported in the literature, but little is known about the biomolecular aspect of MSI stability. Tissue reactions to the applied forces must be known in order to predict the stability of MSIs [8]. Peri-miniscrew implant crevicular fluid (PMICF) is the inflammatory exudate that flows out via the MSI crevice [9]. Biomarkers in the PMICF are used to assess the host’s response to mechanical forces. Like a natural tooth, orthodontic force initiates a cascade of inflammatory reactions around an MSI [8]. PMICF composition is similar to gingival crevicular fluid (GCF) consisting of inflammatory mediators (e.g., cytokines, host-derived enzymes and their inhibitors, antibodies, tissue breakdown products, and host response modifiers), which may show variation during orthodontic force application [9]. The changing expression of biomarkers around an MSI gives some insight into the biological response of peri-implant tissue.

Literature shows several GCF biomarkers for inflammation, tissue damage, bone deposition and resorption, and other biological processes related to the orthodontic tooth movement [10]. However, a limited number of biomarkers have been studied in PMICF regarding MSI stability. IL-1ß [9, 11], IL-2 [12], IL-6 [12], 1 L-8 [12], and TNF-α [13] are pro-inflammatory biomarkers; chondroitin sulphate [14], RANKL/OPG [15], and osteocalcin [16] are bone markers. During inflammation or traumatic insult, ILs and TNF-α act by promoting bone resorption and inhibiting bone formation through the RANK-RANKL pathway, which stimulates endothelial cells, osteoblasts, and osteoclasts. These protein-based biomarkers have shown promising results to help explain the underlying hard tissue response around an MSI during orthodontic loading.

Causative factors in MSI failure (acute/chronic peri-implant inflammation, soft tissue response, and orthodontic loading force) are still an enigma. Circulating cell-free nucleic acids (cfNAs) are nucleic acid-based inflammatory biomarkers that mediate acute inflammation [17, 18] and are present in biological fluids independent of cells. The concentration of cfNAs in healthy individuals tends to be 10–30 ng/ml [19]. A high level of cfNAs is detected in malignant diseases [20]. The cfNAs are also reported as potential biomarkers for Friedreich’s and spinocerebellar ataxias, which are degenerative diseases [21–24]. The main source of cfNAs is apoptosis and necrosis of haematopoietic cells [25]. In response to trauma and acute inflammation, pro-inflammatory cytokines stimulate WBC release (neutrophils) from haematopoietic cells [26]. It is also reported that hyperactivity of neutrophils releases cfNAs in traumatic injury [27–29]. Thus, cfNAs are potential biomarkers to assess inflammatory changes during the insertion and loading of MSIs.

Existing literature describes protein-based biomarkers. However, to the best of our knowledge, there are no reports of cfNAs as biomarkers for MSI stability. Furthermore, there are no studies that compare biomarker (protein or nucleic acid) levels to assess MSI success in PMICF or compare clinical parameters with biochemical parameters.

Therefore, the present study aims to evaluate the changes in the level of cfNAs in PMICF before and after orthodontic force application and to investigate the relationship between the level of cfNAs and the clinical parameters. We hypothesize that there will be a significant difference between the levels of cfNAs in PMICF, before and after orthodontic force application.

## Materials and methods

### Ethical statement

The prospective study was conducted on patients undergoing orthodontic treatment in the Department of Orthodontics and Dentofacial Deformities, All India Institute of Medical Sciences, New Delhi, India. The study received approval from the Institutional Ethics Committee Id: IECPG/32/11/2015.

### Patient selection and study design

There is a paucity of data regarding the relevance of cfNAs as biomarkers in assessing the stability of MSIs. Accordingly, no sample size was calculated a priori, and it was planned that a convenient number of at least 40 implant sites should be studied as an initial investigation to understand the alteration in cfNA levels at different points in time.

The inclusion criteria used in this study were:

- Patients requiring fixed mechanotherapy with first premolar extractions with maximum anchorage requirement
- Patients in the age group 15–20 years, irrespective of sex
- Patients with no history of any systemic disease, hormonal imbalance, or drug intake (antibiotics or anti-inflammatory) during the previous 6 months
- Patients with positive informed consent

**Fig. 1:**
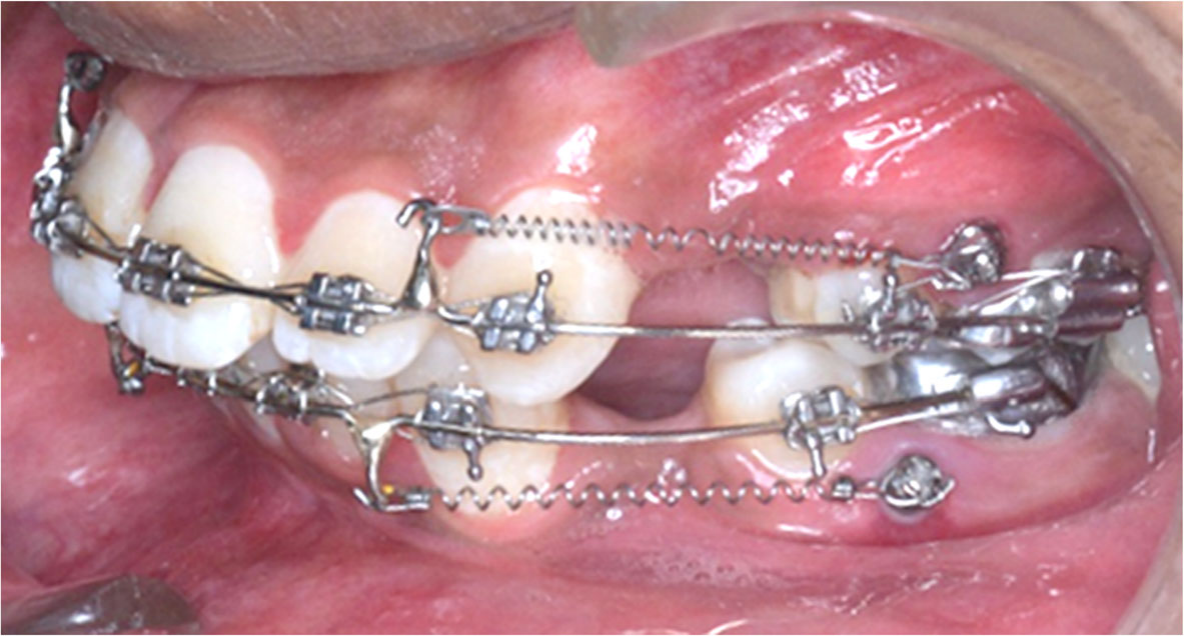
Loading of the miniscrew implant with Nitinol close coil spring (9 mm, 200-g force)

The total sample size consisted of 46 MSIs. Levelling and alignment were achieved till 0.019″ × 0.025″ SS archwire using MBT prescription (Ormco, slot size 0.022 × 0.028) followed by MSI insertion. A self-drill miniscrew implant (8 mm in length, 1.5 mm in diameter, TOMAS MSI-Dentaurum, USA) was inserted bilaterally between the second premolar and first molar for en masse retraction. Direct loading of the MSI was done 3 weeks after insertion with a nickel-titanium closed coil spring (Jaypee, 9 mm in length, 200-g force) (Fig. 1).

**Fig. 2:**
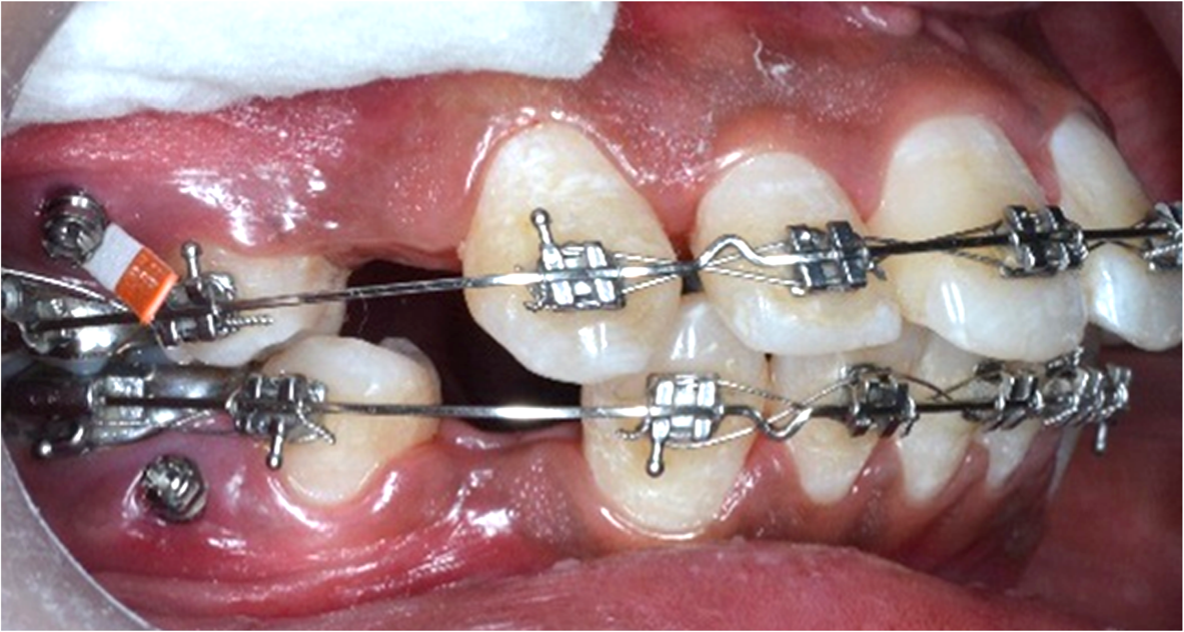
Sample collection by periopaper at the miniscew implant site

**Fig. 3:**
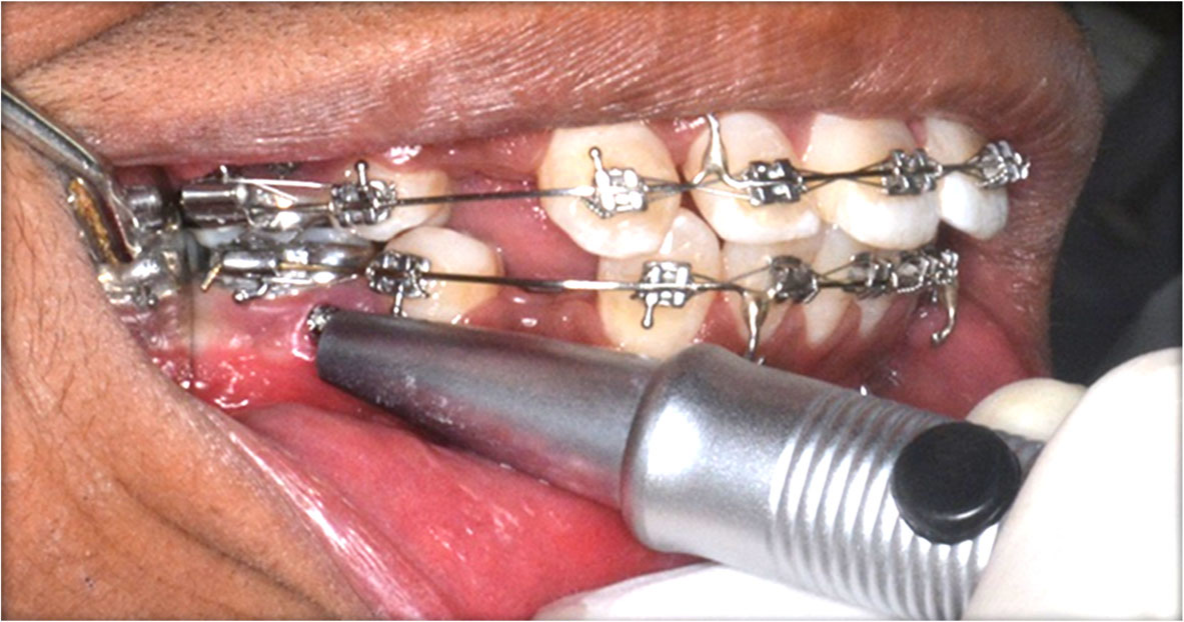
Assessment of mobility of MSI with Periotest

A PMICF sample was collected using Periopaper strips placed at the opening of the peri-miniscrew implant crevice at predetermined intervals (Fig. 2, Table 1). A time interval of 3 weeks after MSI insertion was considered as the baseline parameter since inflammation due to traumatic insertion of implants subsides within 3 weeks [5]. The clinical parameters, including implant mobility and gingival health, were also assessed during the same time interval using a periotest and periodontal probe, respectively (Fig. 3). Gingival health was recorded on a modified Loe and Silness index (gingival index).

**Table 1:** Schedule of sample collection

Loading protocol	CODE	Schedule for sample collection
**Pre-loading**	T1	1 h
	T2	24 h
	T3	3 weeks
**Post-loading**	T4	1 h
	T5	24 h
	T6	7 days
	T7	3 weeks
	T8	6 weeks

### Biochemical analysis

The collected paper strips were eluted in 200 μl of phosphate-buffered saline (PBS) at pH 7.4 to recover cfNAs from the Periopaper strips. After vortexing for 30 s and shaking for a few minutes, soaked paper strips in phosphate buffer solution were kept at 4°C for 12 h to soften the paper and maximize the release of cfNAs. The samples were stored at −70°C until they were assayed for cfNAs.

### Estimation of cfNAs

As per the instruction given by the manufacturer “Invitrogen” HS (High sensitivity DNA). Initially, the Qubit working solution was made followed by 2 standard solutions at 0 = no DNA and 500 pg DNA to calibrate the Qubit fluorometer device. Qubit standard solutions were prepared not more than 2 h prior to the experiment. For quantification, 10 μl of standard and sample was taken with 190 μl of Qubit working solution in 0.5ml PCR tube which was provided with the kit. Vortexing was done for 2-3 s followed by incubation for 2 min at room temperature. After calibration of standards in Qubit 3 device sample was quantified one by one and then each measurement was saved in Qubit 3 device and later, transferred through pen drive.

### Statistical analysis

The cfNA values were expressed as the mean ± SD at each time point. Using a Wilcoxon signed rank test, pre-loading parameters were compared with baseline T1, and post-loading parameters were compared with baseline T3. In addition, the median (interquartile range) was also calculated. The cfNA values were compared between successful and failed MSIs using Wilcoxon’s rank-sum test. A receiver operating characteristic (ROC) curve analysis was carried out to assess the discriminating ability of the markers between the two groups. The best threshold value was identified, and sensitivity, specificity, and predictive values were calculated to predict the failures at the identified threshold values.

Clinical parameters, including periotest mobility, manual mobility, and modified Loe and Silness index, were also assessed. Periotest mobility was compared using Wilcoxon’s rank-sum test. The concordance of pre- and post-loading parameters of the score of severity of gingival inflammation (modified Loe and Silness index) and manual mobility of MSI were assessed by using an exact symmetry test gingival. A *P* value of < 0.05 was considered statistically significant. All analyses were carried out using Stata, version 15.1.

## Results

**Fig. 4:**
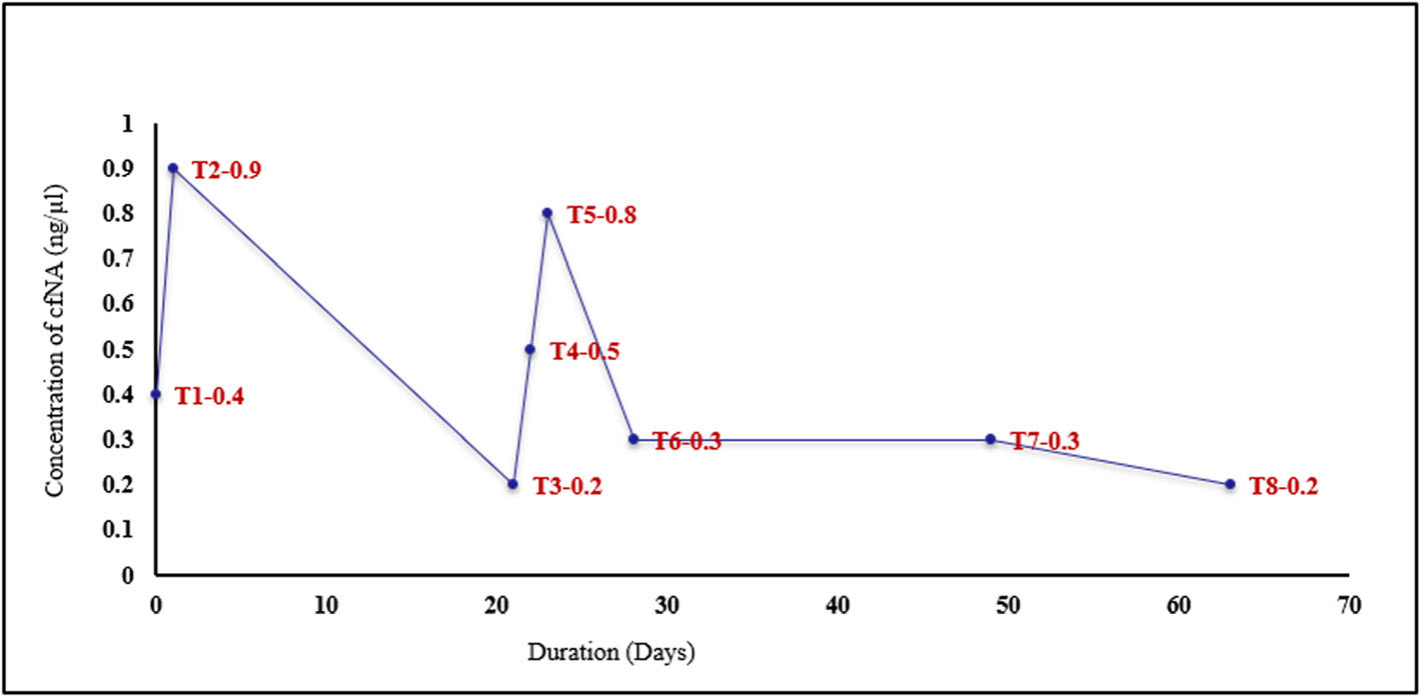
Trend of concentration of cfNAs (ng/μl) at various time intervals in combined all four quadrants

The levels of cfNAs in maxillary and mandibular arches in each patient were tabulated and compared. The trend was found to be the same in each arch, with no statistically significant difference between the two arches. Figure 4 shows the average of the cfNA biomarker level taken for both arches from 12 patients, followed for 63 days.

### Changes in cfNA levels with time (Fig. 4, Table 2)

During pre-loading, there was a significant rise (*P* < 0.01) in the cfNA concentration 24 h after implant insertion (T2, 0.9 ± 0.72 ng/μl). The level of cfNAs significantly decreased over 3 weeks and almost subsided to the baseline level at T3 (0.2 ± 0.31 ng/μl). During post-loading intervals, there was a significant rise (*P* < 0.01) 24 h after MSI loading (0.8 ± 0.70 ng/μl), after which it decreased to 0.2 ± 0.24 ng/μl after 63 days. Thus, the curve of cfNAs showed two peaks, the first at T2 and the second at T5; the T2 peak was higher than the T5 (Fig. 4, Table 2).

**Table 2:** Quantitative levels of cfNAs in PMICF in combined all four quadrants before and after loading of MSI

Loading protocol	Time	Mean (ng/μl)	± SD	Range	***P*** value
**Pre-loading**	T1	0.4	0.86	0.001–4.78	**–**
	T2	0.9	0.72	0.11–2.7	< 0.01
	T3	0.2	0.31	0.001–1.2	0.03
**Baseline for post-loading**	T3	0.2	0.31	0.001–1.2	
**Post-loading**	T4	0.5	0.53	0.12–2.12	0.25
	T5	0.8	0.70	0.04–3.03	< 0.01
	T6	0.3	0.36	0.015–1.42	0.15
	T7	0.3	0.35	0.001–1.2	0.15
	T8	0.2	0.24	0.01–0.93	0.72

*P* < 0.05 = statistically significant, *P* < 0.01 = statistically highly significant

### Correlation of clinical parameters (Table 3)

During pre-loading, there was a significant (*P* < 0.001) rise in the mean periotest value 24 h after MSI insertion (T2, 6.7 ± 3.38), followed by a gradual decrease 3 weeks after MSI insertion (T3, 5.4 ± 2.91). There was a slight surge at T5 (5.4 ± 2.05) after MSI loading and a gradual decrease to 4.1 ± 2.01 after 63 days. When the exact test of symmetry was applied for manual mobility and the modified Loe and Silness index, there was a significant (*P* < 0.001) deterioration in gingival health (65%) and an increase in manual mobility 24 h after implant insertion (Table 3).

**Table 3:** Comparison of periotest mobility, manual mobility (MM), and modified Loe and Silness index before and after loading of MSI

Loading protocol	Periotest mobility			Manual mobility (MM) and Loe and Silness index (LS)		
	Time	Mean ± SD	***P***	Deterioration in MM/LS of implant (%)	Improvement in MM/LS of implant (%)	***P***
**Pre-loading**	T1	3.1 ± 1.67	**–**	–	**–**	
	T2	6.7 ± 3.38	0.00	65	0	< 0.001
	T3	5.4 ± 2.91	0.39	25	30	0.01
**Baseline for post-loading**	T3	5.4 ± 2.91		25	30	
**Post-loading**	T4	5.0 ± 2.40	0.32	25	11.1	0.26
	T5	5.4 ± 2.05	0.05	30.5	0	0.001
	T6	4.4 ± 1.66	0.43	22.2	11.1	0.38
	T7	3.6 ± 2.35	0.97	0.02	19.4	0.07
	T8	4.1 ± 2.01	0.23	16.6	13.9	> 0.90

*P* < 0.05 = statistically significant, *P* < 0.01 = statistically highly significant

### Comparison of cfNA levels between successful and failed MSIs (Table 4)

Among 46 MSIs, 36 were stable during treatment (20 maxilla, 16 mandible, bilaterally), and 10 failed (four maxilla, six mandible, bilaterally). The success rate in the maxilla was 80%, and in the mandible, it was 63%. Out of 10 failed implants, six implants failed 24 h after implant insertion, and four implants failed 3 weeks after implant insertion.

**Table 4:** Comparison of cfNA level (ng/μl) between successful and failed MSI and its ability to predict failed MSI

Time	Success (S) Mean ± SD Median (IQR)	Failure (F) Mean ± SD Median (IQR)	S − F Difference (95% CI)	***P***	AUROC (%) (95% CI)	Cutoff	Sensitivity (%) (95% CI)	Specificity (%) (95% CI)	PPV (%) (95% CI)	NPV (%) (95% CI)
T1	0.4 ± 0.86 0.1 (0.02, 0.31)	0.7 ± 0.36 0.8 (0.49, 1.02)	−0.32 (−0.88, −0.24)	0.05	79.0 (63.8, 94.2)	> 0.4	80 (44.4, 97.5)	86.1 (70.5, 95.3)	61.5 (31.6, 86.1)	93.9 (79.8, 99.3)
T2	0.9 ± 0.72 0.7 (0.32, 1.02)	1.1 ± 0.48 1.2 (0.71, 1.32)	−0.26 (−0.75, 0.22)	0.09	67.9 (51.5, 84.3)	> 1.1	60 (26.2, 87.8)	77.8 (60.8, 89.9)	42.9 (17.7, 71.1)	87.5 (71.0, 96.5)
T3	0.2 ± 0.31 0.1 (0.03, 0.32)	0.9 ± 0.30 0.9 (0.70, 1.06)	−0.65 (−0.98, −0.32)	< 0.01	92.4 (82.0, 100.0)	≥ 0.5	100 (39.8, 100.0)	80.6 (64.0, 91.8)	36.4 (10.9, 69.2)	100.0 (88.1, 100.0)
T4	0.5 ± 0.53 0.3 (0.10, 0.62)									
T5	0.8 ± 0.70 0.7 (0.30, 0.90)									
T6	0.3 ± 0.36 0.2 (0.03, 0.53)									
T7	0.3 ± 0.35 0.1 (0.02, 0.31)									
T8	0.2 ± 0.24 0.04 (0.02, 0.20)									

*IQR* interquartile range, *PPV* positive predictive value, *NPV* negative predictive value, *AUROC curve* Area under the receiver operating characteristic curve

*P* < 0.05 = statistically significant, *P* < 0.01 = statistically highly significant

The cfNA level 1 h after MSI insertion was 0.4 ± 0.86 ng/μl in cases with successful implants compared to 0.7 ± 0.36 ng/μl in cases with failed implants. The expression of cfNA levels was on average 0.32 ng/μl more in the cases with failed implants (*P* 0.05). Similar trends could also be seen 24 h and 3 weeks after MSI insertion. ROC curve analysis indicated that the cfNA levels could discriminate well between successful and failed MSIs. The area under the ROC curve (AUROC) varied from 68% 24 h after MSI insertion to 92% 3 weeks after MSI insertion. One hour after MSI insertion, cfNA levels of > 0.4 ng/μl had a sensitivity of 80% (95% CI 44.4 to 97.5%), specificity of 86% (70.5 to 95.3%), PPV of 61.5% (31.6 to 86.1%), and NPV of 94% (79.8 to 99.3%). Similarly, 24 h after MSI insertion and 3 weeks after MSI insertion, cfNA values of > 1.1 units and > 0.5 units showed good sensitivity (60% and 100%), specificity (78% and 80%), PPV (43% and 36%), and NPV (87% and 100%) (Table 4).

## Discussion

Traditionally, the stability of an MSI was assessed by its mobility and the severity of peri-implant inflammation. However, evolving research on biological markers provides insight into bone remodelling and the health of periodontal and peri-implant tissue. Although a limited number of protein-based biomarkers have been studied in PMICF, the present study is the first to evaluate nucleic acid-based biomarkers during orthodontic tooth movement. This prospective study was initiated with the hypothesis that changes in the clinical stability of MSIs must be reflected in the activity of underlying biomarkers. As age and sex do not affect enzymatic activity [30], these factors were not considered during sample recruitment. The biochemical levels of maxilla and mandible were evaluated separately as the literature supports a difference in the rate of success and failure of MSIs in maxilla and mandible [31]. PMICF volume is correlated with the inflammatory state; therefore, we compared the mean volume of cfNAs at various time intervals (T1–T8).

In the present study, cfNA levels peaked 24 h after MSI insertion; this was statistically significant. The increased level of cfNAs was due to injury to soft tissue and bone, provoking inflammation and cell death. During acute inflammation, cells such as neutrophils, macrophages, and lymphocytes invade the injury site early on, followed by fibroblasts and osteoclasts. Release of cfNAs from necrotic cells occurs during phagocytosis [32]. In addition to cell death, neutrophils can mediate the immune response by releasing neutrophil extracellular traps (NETs) that can trap and kill various pathogens [33]. An increase in the level of NETs has been shown to correlate with the release of cfNAs [34]. These cells and pathogens release cfNAs into the extracellular environment during NETosis, i.e., they are involved in the formation of cfNAs. To the best of our knowledge, very few studies have discussed the peak of biomarkers 24 h after MSI insertion. There was a fall in the level of cfNAs 3 weeks after MSI insertion due to reduced cellular stress, and cell death reduces acute inflammation.

The second peak was observed 24 h after MSI loading due to increased cell proliferation during bone remodelling. However, the level was not as high as that seen 24 h after implant insertion. A slight increase after 24 h indicates that mechanical stress appears to evoke biochemical and structural responses in various cells in vivo and in vitro [35]. Previous studies reported that IL-1β levels significantly increase after 24 and 48 h after MSI loading, suggestive of increased osteoclastic activity during bone remodelling [9, 11]. IL-2 showed a peak at 24 h, and IL-8 showed a peak 1 h after MSI loading [12]. RANKL levels were significantly higher 24 h after MSI loading; correspondingly, the OPG/RANKL ratio decreased, indicating increased osteoclastic activity. TNF-α increased 24 and 48 h after MSI loading, but the increase was not statistically significant. HMG-B1 [36], MMP-8 [37], and pentraxin-3 [38] also peaked 24 h after MSI loading. A recent review also reported that IL-1, IL-2, IL-8, and TNF-α in PMICF, and prostaglandins, OPG/RANKL, and MMP-8 in GCF, are indicators of bone remodelling during orthodontic tooth movement [39].

The cfNA levels decreased over a 6-week period after MSI loading, suggestive of inherent feedback mechanisms and adaptation of periodontal architecture to forces. Existing literature has reported the highest peak at 24 h or 48 h after loading due to underlying bone response to orthodontic forces. Increased inflammation after MSI insertion may be a possible reason for peri-implantitis or implant failure. Based on our findings, we can suggest that cfNAs might play a major role in mediating acute inflammation after MSI insertion.

The highest periotest mobility (7 ± 3) was observed 24 h after MSI insertion. There was a significant increase in manual mobility (65%) and gingival inflammation (65%) 24 h after MSI insertion. Clinical findings correlated with the biochemical findings; the levels of cfNAs also showed a significant rise 24 h after MSI insertion. Conventionally, only clinical parameters have been used to assess the stability of MSIs [40], but emerging studies on biomarkers have opened up new vistas in clinical orthodontics. Biological markers can be assessed and monitored during treatment even before implant failure, so measures such as improvement in oral hygiene and reduction in loading forces can improve MSI stability.

In the present study, a higher failure rate has been observed in the mandible; the survival rate in the maxilla is 80%, but in the mandible is 63%. A higher number of MSI failures in the mandible have also been observed in other studies [41]. In one study, 38 MSIs were placed in 10 patients in both the maxilla and mandible; a success rate of 100% was seen in the maxilla and 76.3% in the mandible [41]. The higher failure rate in human mandibles is attributed to the greater bone density of the mandible, resulting in the use of higher insertion torque values, bone overheating, and less cortical bone formation around the MSI. Another reason for failure could be reduced accessibility for cleaning and retaining saliva due to salivary gland duct opening [42].

In the present study, bilateral MSI failure has been observed. This finding is in line with earlier studies that reported no significant difference in the failure rate of MSI based on insertion site and insertion side (left or right) [43, 44]. This is contrary to another study that reported a slightly higher MSI success rate on the left side, possibly due to better oral hygiene maintenance by a right-handed person [45].

We observed higher levels of cfNAs in cases with failed MSI and it was on an average 0.32 ng/μl more. The elevated level of cfNAs might be associated with increased inflammation-mediated bone resorption around the MSI. One supportive study concluded that different DNA profiles in cell-free GCF could be a potential biomarker of periodontal health and disease [46]. A systematic review also reported that levels of biomarkers in PMICF help in understanding immune-inflammatory peri-implant diseases and in developing host modulation therapies [47]. The present study suggests that an increased level of cfNAs in failed implant cases is a potential indicator of implant stability. Levels of cfNA release, if continuously monitored, can avoid screw loosening. Thus, cfNAs are an important biomarker for anchorage during canine or en masse retraction.

A recent study reported that peptides, such as α defensins, thymosins, trypsin, and antitrypsins, are biological markers essential to understand the biological process behind tooth eruption. It concludes that levels of biomarkers enhance the understanding of the biological basis of tooth movement and tooth eruption [48].

Protein-based biomarkers are estimated using ELISA, whereas nucleic acid-based biomarkers, such as cfNAs, are estimated using fluorometry. Fluorometry is a fluorescent-based technique, simple to perform, and can be done chair-side. It is a non-invasive, rapid, and accurate method of cfNA quantification. The detection of cfNAs is sensitive and specific since the nucleic bases of host DNA and bacterial DNA can be differentiated with this technique. Thus, it plays an important role in determining the causative factors for inflammatory changes, whether the inflammation is exacerbated by the host immune cells or foreign body cells (such as bacteria, viruses, parasites).

The change in the expression of biomarkers indicates the clinical stability of an MSI in orthodontic patients. The conventional methods for detecting these biomarkers still lack accuracy. These conventional methods are time-consuming and require laboratory setups for procedures such as ELISA. Detection of cfNAs by a Qubit 3 device is extremely sensitive and can be done chair-side. Chair-side diagnostic kits also help in assessing the progression of orthodontic MSI stability. It concludes that time-dependent temporal changes of biomarker levels may be of diagnostic value.

Limitations of this study include the small sample size and only one biomarker studied for a short period of time. Additional research is required with a larger sample size for a longer duration to validate the interaction of biomarkers during peri-implantitis. Studies need to be conducted in GCF and PMICF simultaneously to find out optimal forces for tooth movement. Real-time PCR should be carried out to determine whether the cause of implant failure is host DNA or bacterial DNA.

## Conclusion

The highest peak in the level of cfNAs was observed 24 h after MSI insertion. The expression of the level of cfNAs was greater in cases with a failed MSI. The cfNAs play a major role in mediating acute inflammation. Therefore, cfNAs are an important prognostic biomarker to assess implant stability and the health of soft and hard tissues around an MSI.

## Data Availability

The patients and the material were provided by the institute after the approval by the corresponding author.

## References

[1] Park JH, Shin K (2020). An overview of clinical applications for temporary anchorage devices. Temporary anchorage device in clinical orthodontics.

[2] Ntolou P, Tagkli A, Pepelassi E (2018). Factors related to the clinical application of orthodontic mini-implants. J Int Oral Health.

[3] Antoszewska-Smith J, Sarul M, Łyczek J, Konopka T, Kawala B (2017). Effectiveness of orthodontic miniscrew implants in anchorage reinforcement during en-masse retraction: a systematic review and meta-analysis. Am J Orthod Dentofac Orthop.

[4] Creekmore TD, Eklund MK (1983). The possibility of skeletal anchorage. J Clin Orthod.

[5] Kharbanda OP, Kapoor P, Rajeswari MR, Kapila SD, KWL V, Huang GJE (2017). Peri-miniscrew biomarkers as indicators for miniscrew stability or failure. Anecdote, expertise and evidence: applying new knowledge to everyday orthodontics, Craniofacial growth series.

[6] Kanomi R (1997). Mini-implant for orthodontic anchorage. J Clin Orthod.

[7] Schatzle M, Mannchen R, Zwahlen M, Lang NP (2009). Survival and failure rates of orthodontic temporary anchorage devices: a systematic review. Clin Oral Implants Res.

[8] Kaur A, Kharbanda OP, Kapoor P, Kalyanasundaram D (2017). A review of biomarkers in peri-miniscrew implant crevicular fluid (PMICF). Prog Orthod.

[9] Monga N, Chaurasia S, Kharbanda OP, Duggal R, Rajeswari MR (2014). A study of interleukin 1β levels in peri-miniscrew crevicular fluid (PMCF). Prog Orthod.

[10] Kapoor P, Kharbanda OP, Monga N, Miglani R, Kapila S (2014). Effect of orthodontic forces on cytokine and receptor levels in gingival crevicular fluid: a systematic review. Prog Orthod.

[11] Sari E, Ugar C (2007). Interleukin 1beta levels around microscrew implants during orthodontic tooth movement. Angle Orthod.

[12] Hamamcı N, Acun Kaya F, Uysal E, Yokuş B (2012). Identification of interleukin 2, 6, and 8 levels around miniscrews during orthodontic tooth movement. Eur J Orthod.

[13] Kaya FA, Hamamcı N, Uysal E, Yokuş B (2011). Identification of tumor necrosis factor-α levels around miniscrews during canine distalization. Kor J Orthod.

[14] Intachai I, Krisanaprakornkit S, Kongtawelert P, Ong-chai S, Buranastidporn B, Suzuki EY, Jotikasthira D (2010). Chondroitin sulphate (WF6 epitope) levels in peri-miniscrew implant crevicular fluid during orthodontic loading. Eur J Orthod.

[15] Enhos S, Veli I, Cakmak O, Ucar FI, Alkan A, Uysal T (2013). OPG and RANKL levels around miniscrew implants during orthodontic tooth movement. Am J Orthod Dentofac Orthop.

[16] Dhanashetti R, Dharma RM, Dinesh MR, Amarnath BC, Prashanth CS, Shetty A (2016). Evaluation of osteocalcin as a biomarker to determine implant stability. Ind J Stomatol.

[17] Pos O, Biro O, Szemes T, Nagy B (2018). Circulating cell-free nucleic acids: characteristics and applications. Eur J Hum Genet.

[18] Swarup V, Rajeswari MR (2007). Circulating (cell-free) nucleic acids – a promising, non-invasive tool for early detection of several human diseases. FEBS Lett.

[19] Steinman CR (1975). Free DNA in serum and plasma from normal adults. J Clin Invest.

[20] Leon SA, Green A, Yaros MJ, Shapiro B (1975). Radioimmunoassay for nanogram quantities of DNA. J Immunol Methods.

[21] Srivastava AK, Padma MV, Gulati S, Rajeswari MR (2019). Quantitative proteomic and network analysis of differentially expressed proteins in PBMC of Friedreich’s ataxia (FRDA) patients. Front Neurosci.

[22] Dantham S, Srivastava AK, Gulati S, Rajeswari MR, Pathak D (2018). Differentially regulated cell-free miRNAs in the plasma of Friedreich’s ataxia and their association with disease pathology. Neuropediatrics..

[23] Danthum S, Srivastava AK, Gulati S, Rajeswari MR (2016). Plasma circulating cell-free mitochondrial DNA in the assessment of Friedreich’s ataxia. J Neurol Sci.

[24] Rajeswari MR, Moganty SV, Srivastava AK, Padma MV (2016). Non-invasive biomarkers for spinocerebellar ataxias, types 2 & 12 patients and their correlation with cerebellar degeneration. Parkinsonism Relat Disord.

[25] Lui YY, Chik KW, Chiu RW (2002). Predominant hematopoieticorigin of cell-free DNA in plasma and serum after sex-mismatched bone marrow transplantation. Clin Chem.

[26] Pietras EM (2017). Inflammation: a key regulator of hematopoietic stem cell fate in health and disease. Blood..

[27] Margraf S, Logters T, Reipen J, Altrichter J, Scholz M, Windolf J (2008). Neutrophil-derived circulating free DNA (cf-DNA/NETs): a potential prognostic marker for posttraumatic development of inflammatory second hit and sepsis. Shock..

[28] Rygh P, Selvig KA (1973). Erythrocyte crystallization in rat molar periodontium incident to tooth movement. Scand J Dent Res.

[29] Storey E (1973). The nature of tooth movement. Am J Orthod.

[30] Serra G, Morais LS, Elias CN, Meyers MA, Andrade L, Müller CA, Müller M (2010). Sequential bone healing of immediately loaded mini-implants: histomorphometric and fluorescence analysis. Am J Orthod Dentofac Orthop.

[31] Lin Y-D, Wu Y-K (2015). Factors affecting the stability of mini-implants. Chin J Tissue Eng Res.

[32] Jahr S, Hentze H, Englisch S, Hardt D, Fackelmayer FO, Hesch RD, Knippers R (2001). DNA fragments in the blood plasma of cancer patients quantitation and evidence for their origin from apoptotic and necrotic cells. Cancer Res.

[33] Thierry AR, El Messaoudi S, Gahan PB (2016). Origins, structures, and functions of circulating DNA in oncology. Cancer Metastasis Rev.

[34] Fuchs TA, Kremer Hovinga JA, Schatzberg D, Wagner DD, Lämmle B (2012). Circulating DNA and myeloperoxidase indicate disease activity in patients with thrombotic microangiopathies. Blood..

[35] Tzannetou S, Efstratiadis S, Nicolay O, Grbic J, Lamster I (1999). Interleukin-1β and β-glucuronidase in gingival crevicular fluid from molars during rapid palatal expansion. Am J Orthod Dentofac Orthop.

[36] Ilancheralathan C. Quantitative evaluation of HMGB1 protein level around miniscrew site during orthodontic tooth movement [MDS Orthodontics Thesis]. AIIMS. 2015.

[37] Negi N. The levels of MMP-8 around miniscrew implant site during orthodontic tooth movement [MDS Orthodontics Thesis]. AIIMS. 2016.

[38] Machawal J. Quantitative evaluation of Pentraxin-3 in peri-miniscew implant crevicular fluid before and after orthodontic force application [MDS Orthodontics Thesis]. AIIMS. 2018.

[39] Kumar SM, Saba I, Bano N, Mathew S (2019). Biomarkers in peri mini screw implant crevicular fluid in orthodontics - a review. JDER..

[40] Sakin C, Aylikci O (2013). Techniques to measure miniscrew implant stability. J Orthod Res.

[41] Samrit V, Kharbanda OP, Duggal R, Seith A, Malhotra V (2012). Bone density and miniscrew stability in orthodontic patients. Aust Orthod J.

[42] Papageorgiou SN, Zogakis IP, Papadopoulos MA (2012). Failure rates and associated risk factors of orthodontic miniscrew implants: a meta-analysis. Am J Orthod Dentofac Orthop.

[43] Motoyoshi M, Yoshida T, Ono A, Shimizu N (2007). Effect of cortical bone thickness and implant placement torque on stability of orthodontic mini-implants. Int J Oral Maxillofac Implants.

[44] Chuang SK, Tian L, Wei LJ, Dodson TB (2001). Kaplan-Meier analysis of dental implant survival: a strategy for estimating survival with clustered observations. J Dent Res.

[45] Beltrami R, Sfondrini F, Confalonieri L, Carbone L, Bernardinelli (2015). Miniscrews and mini-implants success rates in orthodontic treatments: a systematic review and meta-analysis of several clinical parameters. Dentistry.

[46] Thaweboon B, Laohapand P, Amornchat C, Matsuyama J, Sato T, Nunez PP, Uematsu H, Hoshino E (2010). Host beta-globin gene fragments in crevicular fluid as a biomarker in periodontal health and disease. J Periodontal Res.

[47] Dursun E, Tozum TF (2016). Peri-implant crevicular fluid analysis, enzymes and biomarkers: a systemetic review. J Oral Maxillofac Res.

[48] Lavarone (2020). Top down proteomic analysis of gingival crevicular fluid in deciduous, exfoliating and permanent teeth in children. J Proteome.

